# Vacuum-Assisted Closure of Perineal War Wound Related to Rectum

**Published:** 2009-11-13

**Authors:** Nazim Gümüş

**Affiliations:** Plastic and Reconstructive Surgery Department, Adana Numune Research and Training Hospital, Adana, Turkey

## Abstract

**Introduction:** Vacuum-assisted wound closure therapy has widely been used in various clinical applications with successful results and has considerably increased in popularity over the past decade. The patient who sustained a complex war wound to his perineum has been presented. **Methods:** After the initial treatment he was discharged from the hospital in which he had been treated for 4 days in Iraq. On the examination, all wounds were deeply contaminated with foreign bodies and also involved significant volume of devitalized tissue. Perineal injury had not only caused a large skin defect but also left a deep wound leading to rectal perforation, so the wound and its borders were quite contaminated and infected by rectal contents. After improving his general condition with medical treatment, he underwent an immediate operation in which first a colostomy was performed and then the wound tract placed between perineum and rectum was sharply and extensively debrided to viable-appearing bleeding tissue to remove the whole necrotic tissues, foreign bodies, cloths, and debris. At the end of the intervention, a negative pressure dressing was applied and used during 12 days and then completed. **Results:** The wound tract obliterated entirely without permitting any leakage of rectal contents, and wound bed appeared clean, granulated, contracted, and viable enough for definitive closure with flap mobilization. **Conclusions:** When dealing with this experience presenting an unusual wound that was in a very difficult area of the body for the treatment, perineum, caused from a challenging reason, war injury, and also was complicated with rectal injury, the technique seems to have a significant beneficial effect on the healing of complicated wounds such as in perineal wound and war wound, even if these are at risk of severe infection and progressive tissue necrosis.

Vacuum-assisted wound closure therapy has widely been used in various clinical applications with successful results and has considerably increased popularity over the past decade. Its indications have been expanding constantly with time since it was first introduced in clinical use, which includes pressure sores, stasis ulcers, chronic wounds such as diabetic foot ulcers, posttraumatic and postoperative wounds, infected wounds such as necrotizing fasciitis or sternal wounds, soft-tissue injuries, injuries with bone exposure, and open abdominal wounds.^1^–^8^ Recently, it has been used successfully for war wounds to provide early closure without existing any signs of infection.^9^–^11^

Effects of vacuum-assisted closure (VAC) therapy on wound healing mainly consist of increasing granulation tissue formation rapidly and making bacterial clearance with a suction force removing excess fluid and debris that leads to a decrease in tissue bacterial levels and an increase in tissue perfusion that results in improved wound healing.^4^,^8^ Actually, the therapy is not used instead of surgical debridement, but it has an important complementary function in the wound healing, so in the initial treatment of every contaminated wound or tissue necrosis, either surgical necrosectomy or debridement should be considered as a first choice. All wounds treated with VAC device must be followed carefully against wound sepsis and, if necessary, surgical debridement should be performed extensively while dressing is changed.

The case of a patient with a complex war wound on his perineum, who was treated successfully with VAC after coming from Iraq, is presented here.

## CASE REPORT

A 54-year-old man with a 5-day history of injury on his left arm, lumbar area, and perineum due to the explosion of a bomb in Iraq was admitted to our clinic. After the initial treatment he was discharged from the hospital in which he had been treated for 4 days. On the first examination, all wounds were deeply contaminated by mess and foreign bodies and also involved significant volume of devitalized tissue. Moreover, the patient was dehydrated and pyrexic (38.9°C). Injuries involving left arm and lumbar area had affected almost entire skin and subcutaneous tissue resulting in soft tissue defect, but perineal injury had not only caused a large skin defect but also penetrated into the rectum leading to perforation, so the wound and its adjacent area were quite contaminated and infected by rectal contents.

**Figure 1:**
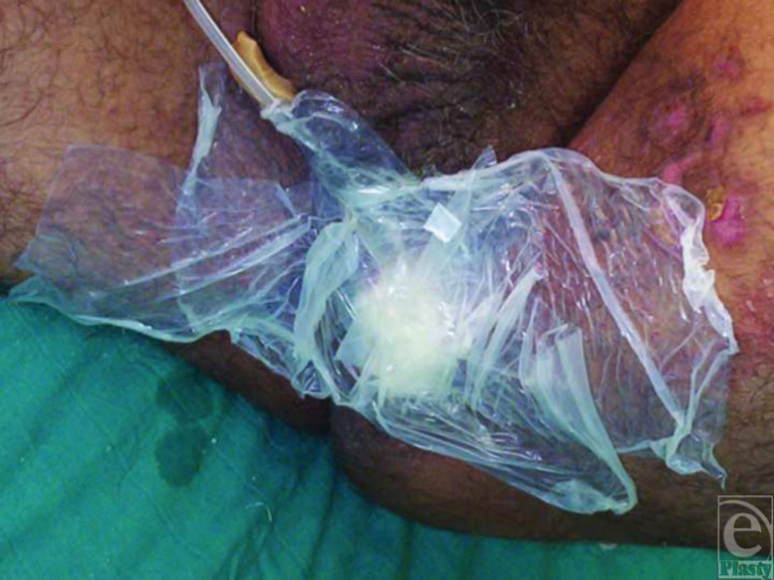
Appearance of the vacuum-assisted closure dressing placed after the extensive debridement of the perineal war wound.

**Figure 2:**
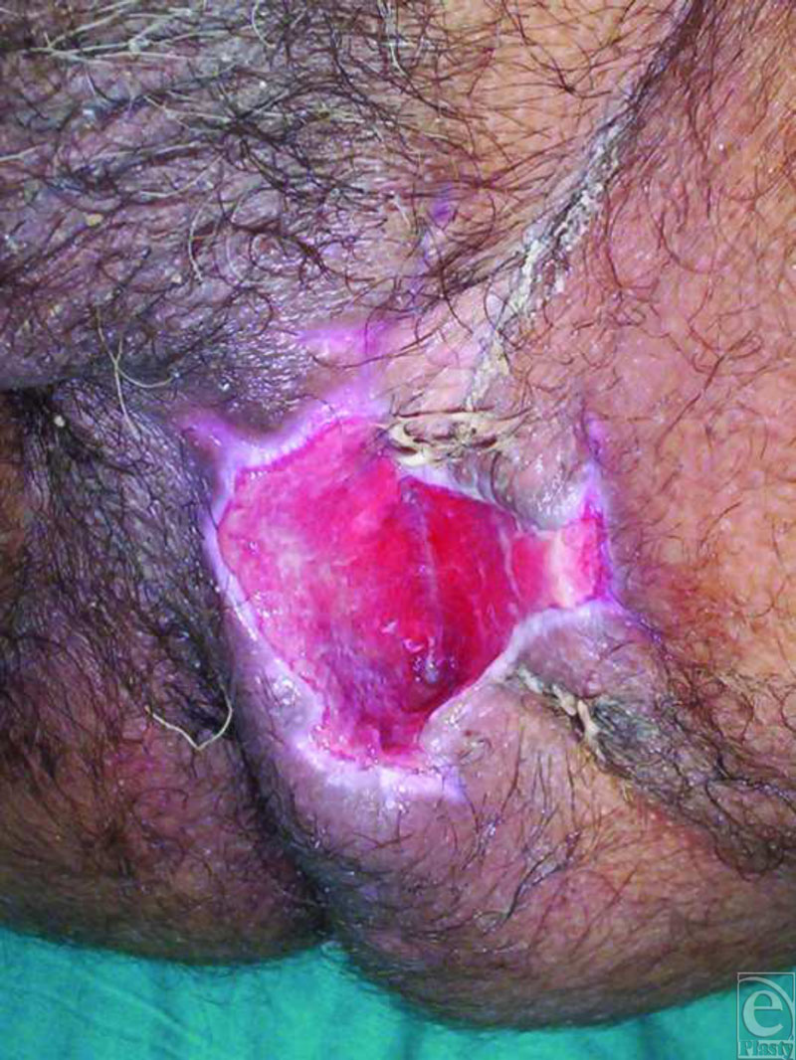
View of the wound on the eighth day after the beginning of the vacuum-assisted closure therapy. Note that some granulation tissue became apparent and the wound tract obliterated and contracted on the base of the wound.

**Figure 3:**
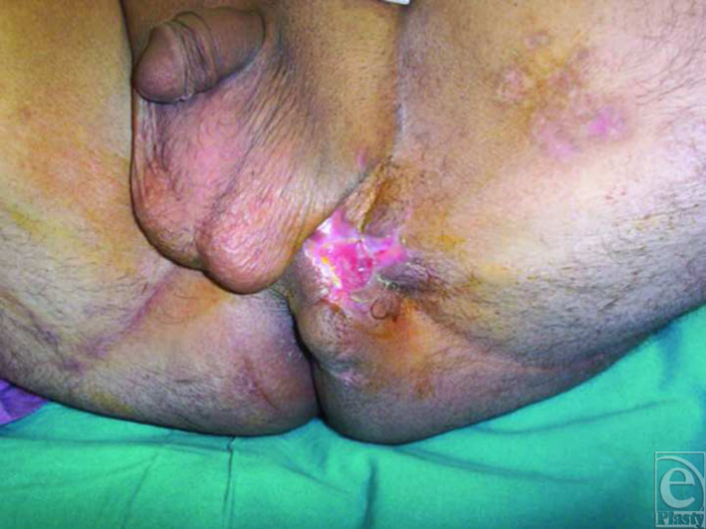
Appearance of the wound on the 13th day after starting the therapy, showing that it almost entirely contracted, and the tract obliterated completely without permitting any leakage of rectal contents.

**Figure 4:**
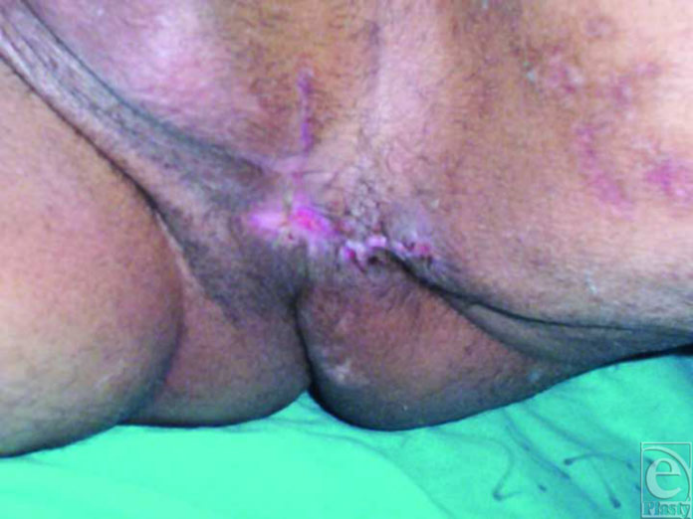
Appearance of the perineal area 10 days later the closure with flap mobilization.

After improving his general condition with medical treatment including fluid replacement, intravenous antibiotics, analgesics, and antipyretics, he underwent an immediate operation in which first a colostomy was performed to stop constant contamination of the perineal wound by the rectal content, and then perineal wound was lavaged with about 2-L sterile saline solution using jet irrigation, and cleansed with antiseptic solutions. Later, the wound tract placed between perineum and rectum was sharply and extensively debrided to viable-appearing bleeding tissue with meticulous hemostasis to remove the whole necrotic tissues, foreign bodies, cloths, and debris. At the end of the intervention, a negative pressure dressing consisting of a black polyurethane foam with 400- to 600-µm pores and transparent adhesive drape was used to cover the complex perineal wound and set to 125 mm Hg continuous pressure (Fig 1). Then, it was left in place for 3 days with daily examination of closed wound for any signs of infection or other local complications. In each dressing change under sterile conditions, additional lavage and debridement were performed, if necessary. Vacuum-assisted closure therapy was used during 12 days and then completed, as the wound tract obliterated entirely without permitting any leakage of rectal contents, and wound bed appeared clean, granulated, contracted, and viable, suitable for definitive closure with flap mobilization (Fig 2). In the second operation, the entire wound was closed by using local flaps without encountering any difficulty (Fig 3). In the follow-up, no complication was observed (Fig 4).

## DISCUSSION

Vacuum-assisted closure therapy has been used successfully in the treatment of challenging wounds such as open abdomen, large burns, degloving injuries, infected pilonidal sinuses, defects resulting from abdominal peritoneal resections and pelvic exenterations, sternal infections with sternal instability, and mediastinitis in which dressing changes are difficult and have high risk of contamination. Perineal area is one of the most special regions for securing the wound dressing and avoiding bacterial contamination to grow uneventful wound healing, so VAC dressing may be considered to be the best choice for overcoming these problems in the treatment. The technique allows continuous drainage from the contaminated or complicated wounds with closed dressing in safety, facilitating the reduction of the bacterial levels, particles, debris, and exudate. Also it is capable of removing several liters of fluid in either large complicated or deep wounds.^3^,^4^,^8^ In the present case, perineal dressing was secured constantly by subatmospheric pressure without existing any problem, also fluid coming from the wound was drained completely by negative pressure, leading to collapse of the wound cavity in few days, although rectal communication and some tissue necrosis presented.

Bronchard et al^6^ have used successfully VAC therapy in the treatment of perineal necrotizing skin and soft tissue infections of 6 patients who had extensive, deep, and life-threatening infections. With a median number of 6 dressings, complete granulation of the wound developed, which allowed skin closure and reconstructive surgery.^6^ Unlike their cases, our patient had a perineal wound related to the rectum due to an explosion in the war and seemed to have a more complex wound; however, complete closure was achieved using only 4 dressings in 12 days without needing any skin grafting. Schaffzin et al^1^ presented 4 cases of complex perineal wounds due to wide debridement of severe hidradenitis and Fournier's gangrene, excision of mucinous adenocarcinoma of the anal canal, and abdominoperineal resection. After using VAC device for wound care, they have emphasized that wound care was simplified and healing accelerated, which led to earlier wound closure, early skin grafting with improved graft adherence, and earlier hospital discharge.^1^ When corresponding to our experience, similar outcomes were obtained although our patient had a war wound.

Leininger et al^9^ reported their experiences with wound VAC in war injuries that are frequently high-energy wounds that involve a large amount of devitalized and contaminated tissues, with high risk of infection and wound complications. After performing aggressive debridement and pulsatile lavage, war wounds were covered with negative pressure dressings. When appearing to be clean and viable, these were definitively closed by delayed primary closure, flap mobilization, or split-thickness skin grafting.^9^ Their results are very interesting and successful; in addition, they had neither any infection nor other complication. Although we have presented a case of a war injury penetrating from perinea through the rectum that seems to have a greater risk of infection in the course of the treatment, our outcomes are very similar to the findings of Leininger et al.^9^

When dealing with this experience presenting an unusual wound that placed in a very difficult area of the body for the treatment, perineum, caused from a challenging reason, war injury, and also was complicated with rectal injury, the technique seems to have a significant beneficial effect on the healing of complicated wounds such as perineal and war wounds, even if they are at risk of severe infection and progressive tissue necrosis.
